# Ten Simple Rules for a Computational Biologist’s Laboratory Notebook

**DOI:** 10.1371/journal.pcbi.1004385

**Published:** 2015-09-10

**Authors:** Santiago Schnell

**Affiliations:** 1 Department of Molecular and Integrative Physiology, University of Michigan Medical School, Ann Arbor, Michigan, United States of America; 2 Department of Computational Medicine and Bioinformatics, University of Michigan Medical School, Ann Arbor, Michigan, United States of America; 3 Brehm Center for Diabetes Research, University of Michigan Medical School, Ann Arbor, Michigan, United States of America; Accelrys, UNITED STATES

One of the major hurdles I face as the head of a computational biology laboratory is convincing my research team—particularly those pursuing exclusively mathematical and computational modeling—that they need to keep a laboratory notebook. There seems to be a misconception in the computational biology community that a lab notebook is only useful for recording experimental protocols and their results. A lab notebook is much more than that. It is an organizational tool and memory aid, which serves as the primary record of scientific research and activity for all scientists. It also serves as a legal record of ownership of the ideas and results obtained by a scientist. Here, I present the best practices (summarized as ten rules) for keeping a lab notebook in computational biology, for scientists pursuing exclusively “dry” research.

## Rule 1: Learn Your Institution’s or Laboratory’s Notebook Policy

A lab notebook is an important tool for good record-keeping, research management, to protect intellectual property and prevent fraud [1]. Leading research institutions, research and development divisions in companies, and universities have comprehensive lab notebook policies, which research laboratories should implement. In the absence of an institutional policy, your research group should have a policy to explain to all team members the process for daily record-keeping and maintaining laboratory records. If your institution or laboratory does not have a standard policy, the following rules provide you with some guidelines for keeping a record of your scientific activities—a record that will likely be very important for you, your research supervisor, or peers.

## Rule 2: Select the Right Medium for Your Lab Notebook

There are three types of lab notebooks: the bound or stitched notebook, the loose-leaf or three-ring binder notebook, and the computer-based electronic notebook [1]. Each of these notebooks has its advantages and disadvantages [2]. You will need to select the right type of notebook for your research. Most computational biologists work on several projects at the same time. If you find it too complicated to keep all of your projects in a single lab notebook, you can maintain a lab notebook for each project. Alternatively, you can use a ring binder, in which each project is maintained behind a separate tab divider inside the binder. You also have the option of using an electronic lab notebook. The advantage of electronic notes is that they can be searched easily [3], and computer-generated figures can be quickly copied to the notebook. If you do not identify the right technology, it can be very time-consuming to make a polished electronic notebook. Some laboratories [4] are writing lab notebooks using Microsoft Word, saving as “Web Page,” and automatically transfer the entries into a WordPress.com blog. Microsoft OneNote is a proprietary software option. If you write a lot of computer code and collect large datasets, the electronic notebook will be a better option for you because you can store and link your code and data to your electronic lab records [5]. Electronic lab notebooks can also be shared and accessed easily online with collaborators and lab members [3,4]. However, if you have not identified the right technology for an electronic lab notebook, then you should organize your computer code and data in a safe medium (e.g., hard drive, CD-ROM, cloud storage, version-control databases, paper copies) [6] and record in your notebook where the code or data can be found.

## Rule 3: Make the Habit of Keeping the Lab Notebook in Your Desk

You need to keep your lab notebook at hand, and write things down while you are working. If you rely on your recollection to remember a good idea, a suggestion during a meeting, or an important step in your data analysis or model simulation, you can find yourself in a situation where you no longer remember that critical thought. Furthermore, you will find that writing provides you the opportunity to reflect on these ideas as you put together a logical argument supporting your conjectures, results, or conclusions.

## Rule 4: Record All Scientific Activities in Your Lab Notebook

There are scientists who believe that lab notebook records should be limited to wet or dry lab experimental entries. However, the intellectual activity of a theoretical scientist is not limited to experiments to test hypotheses. Thinking about the possible directions of your research and theorizing about how a system works is often how scientific breakthroughs are made. If you use paper pads or other assorted pieces of paper to write down your ideas or take notes during meetings and seminars, you may lose important items or waste too much time looking for ideas later. Recording your scientific activities will solve this problem. Scientists should record every experiment, every result, every research meeting, notes from seminars, research conference calls, thoughts related to their research problem—all of these items go into their lab notebooks. In a very real way, the lab notebook is a chronological log of everything scholarly a scientist does. Each lab notebook entry should be written immediately after the activity or work was performed.

## Rule 5: Every Entry Should Be Recorded with a Date, Subject, and Protocol

The most logical organization of a lab notebook is chronological. Each entry should contain the date it was made and subject of the entry. If you make distinct sets of entries in the same day, you should separate them by using heading titles and leave sufficient space between the entries [1]. The titles of your entries are important. They should be short, sharp, and informative, as you will use them to build a table of contents for your lab notebook. If you are using a paper lab notebook, you will need to write the date and time stamp and make your entries legible, written in permanent ink and in a language accessible to everyone in the laboratory. If you use an electronic lab notebook, the date and time stamps will be entered automatically for each new subject.

You should also include a brief background for each entry [2]. It could be a short synopsis of your thought process that explains why this entry is important for your scientific work. You can support it with references published in the literature, ideas learned from a research talk, or a previous model developed by your laboratory. Then, record everything you do for this specific entry. If you make a mistake in your paper lab notebook, put a thin line through the mistake and write the new information next to it [1]. Never erase or destroy an entry, because errors or mistakes are an important part of the scientific process.

## Rule 6: Keep a Record of How Every Result Was Produced

The gold standard of science is reproducibility [7]. You need to keep a record of how every result was produced in your in silico experiments, statistical analyses, and mathematical or computational models. Noting the sequence of steps taken allows for a result or analysis to be reproduced. For every step in your model, analysis, or experiment, you should record every detail that will influence its execution [8]. This includes the preparation of your wet or dry experiment, preprocessing, execution with intermediate steps, analysis, and postprocessing of results [1,2]. You should also store the raw data for every figure. This will allow you to have the exact values for the visualization of your results. It will also give you the opportunity to redraw figures to improve their quality or ensure visual consistency for publication.

If a result is obtained through a mathematical model, algorithm, or computer program, you need to include the equations and name and version of the algorithm or computer program, as well as the initial conditions and parameters used in the model. In many instances, a statistical or computational analysis creates intermediate data [9]. You should record intermediate results because they can reveal discrepancies with your final results, particularly if your analysis is time consuming or readily executable. At the same time, they can help you track any inconsistencies in your analysis or algorithms [8]. Electronic lab notebooks can be very convenient for storing data and linking to computer mathematical models, algorithms, and software stored in cloud mass storage systems.

## Rule 7: Use Version Control for Models, Algorithms, and Computer Code

As a mathematical and computational biologist, you will be updating your models, algorithms, or computer programs frequently. You will also create scripts containing initial conditions and parameters to run analyses or simulations. Changes in your models, algorithms, programs, or scripts could drastically change your results. If you do not systematically archive changes, it will be very difficult or impossible to track the codes that you used to generate certain results [8,9]. Nowadays, there are version control systems to track the evolution of algorithms and computer programs or changes in scripts. Bitbucket, Git, Subversion, and Mercurial are among the most widely used version-control systems. You should use a standardized name system to identify changes. If you have a paper lab notebook, you should record the name and location of the scripts. Those using electronic lab notebooks can add links to each version of their scripts or programs.

## Rule 8: Keep a Lab Notebook That Can Serve As a Legal Record of Your Work

The lab notebook serves as a legal record of ownership of ideas and results [10]. Lab notebooks can serve to determine authorship in scientific papers or rights for establishing copyright or patent rights. If you do not feel comfortable walking around with a notebook or having more than one lab notebook, you can still record all your notes in paper pads, but you should file them in a ring binder using the indexing system of a lab notebook. You can also use a tablet if you keep an electronic lab notebook. However, this is not generally advisable. At the moment, bound notebooks with numbered pages are the only legally recognized option to record and protect your work. Electronic records can be printed out on a regular basis and then bound to form a legally recognized laboratory notebook. If you keep a loose-leaf lab notebook, you should have a parallel hardbound notebook summarizing progress on your projects as a legal record of your work. For each lab notebook entry, clearly indicate who did what work and who was present for a discussion or in silico experiment; this is particularly important for collaborative projects. In addition, each entry should be signed by you and cosigned by a coworker or supervisor. Otherwise, the entry will not serve as a legally valid record.

It is important to note that lab notebooks can be subpoenaed as evidence in cases of scientific misconduct by your institution or governmental funding agencies. According to educational material provided by the Office of Research Integrity at the United States Department of Health and Human Services and the University of New Hampshire, “The integrity of research and scholarly activities depends on accurate, detailed, organized, complete, and accessible data” [11]. From the legal point of view, you keep a lab notebook to reflect your own scientific integrity and to give others the opportunity to corroborate the results of your research. If you keep an electronic lab notebook, you can make it open to the public. This will make your work more transparent to the scientific community, and it will give the opportunity for other scientists to learn more about your research, including the experiments and models that did not work out.

## Rule 9: Create a Table of Contents for Your Lab Notebook

You should record the titles of all entries in your lab notebook in a table of contents as you finish each entry or day. The idea of this index is to help you, your research supervisor, or someone else find the record of your scientific work efficiently [1,2]. There are multiple formats for the table of contents. You should use the format agreed upon in your laboratory. It is generally advisable that each entry in the table of contents has the date the entry has been made, the subject of your entry, and where in the lab notebook the entry can be found. To find information easily in your lab notebook, you should always start an event in a new page. Then, label the event accordingly ("Research Review with Dr. Williams," "Seminar by Dr. Murray," "Thoughts on Project X," etc.) and date it. Once you have done this, you can start taking notes and take as many pages as you need. If you have the standard paper lab notebook, the pages will be numbered. Now you will be in the position to move forward with the critical step: create a table of contents on the first page of your notebook, where you will log the event and page number. If you do not have the standard lab notebook, you can put a page number at the top of each pair of pages counting by two. An advantage of electronic lab notebooks is that you do not need to worry about creating a table of contents because it will be done automatically for you.

## Rule 10: Protect Your Lab Notebook

As your research activities are funded by or through your academic institution, your lab notebook does not belong to you; it belongs to your institution [1,10]. Your lab notebook is part of the scientific legacy of your laboratory. Therefore, you need to protect your lab notebook. Paper lab notebooks should not be taken home. When you leave the laboratory each day, you should leave your lab notebook in a location where your research supervisor can find it. Ideally, you should lock it in the same place every day. If your research institution or lab supervisor allows, you will be able to make copies of your lab notebook, but the original belongs to the institution that paid your salary and handled your research funds.

Laboratories, research institutes, and universities have archival systems in operation to store lab notebooks. You might find this surprising, but lab notebooks generally end up in your university or research institution’s library. Maybe one day you will become a very important scientist, and your lab notebook will be shown in an exhibit, like Charles Darwin’s Notebook B, sketching the relationships between related species, or Albert Einstein’s notebook from 1928, elaborating how the Einstein field equations (a set of ten equations in his theory of general relativity) can be extended to explain electricity and magnetism.
